# Laser-plasmas in the relativistic-transparency regime: Science and applications

**DOI:** 10.1063/1.4983991

**Published:** 2017-05-30

**Authors:** Juan C. Fernández, D. Cort Gautier, Chengkung Huang, Sasikumar Palaniyappan, Brian J. Albright, Woosuk Bang, Gilliss Dyer, Andrea Favalli, James F. Hunter, Jacob Mendez, Markus Roth, Martyn Swinhoe, Paul A. Bradley, Oliver Deppert, Michelle Espy, Katerina Falk, Nevzat Guler, Christopher Hamilton, Bjorn Manuel Hegelich, Daniela Henzlova, Kiril D. Ianakiev, Metodi Iliev, Randall P. Johnson, Annika Kleinschmidt, Adrian S. Losko, Edward McCary, Michal Mocko, Ronald O. Nelson, Rebecca Roycroft, Miguel A. Santiago Cordoba, Victor A. Schanz, Gabriel Schaumann, Derek W. Schmidt, Adam Sefkow, Tsutomu Shimada, Terry N. Taddeucci, Alexandra Tebartz, Sven C. Vogel, Erik Vold, Glen A. Wurden, Lin Yin

**Affiliations:** 1 Los Alamos National Laboratory, P.O. Box 1663, Los Alamos, New Mexico 87545, USA; 2 Department of Physics and Photon Science, Gwangju Institute of Science and Technology (GIST), Gwangju 61005, South Korea; 3 Physics Department, University of Texas at Austin, Austin, Texas 78712, USA; 4 Institute for Nuclear Physics, Technical University of Darmstadt, 64289 Darmstadt, Germany; 5 Institute of Physics of the ASCR, ELI-Beamlines, 182 21 Prague 8, Czech Republic; 6 Spectral Sciences, 4 Fourth Avenue, Burlington, Massachusetts 01803, USA; 7 Laboratory for Laser Energetics, Rochester, New York 14623, USA; 8 Sandia National Laboratories, Albuquerque, New Mexico 87185, USA

## Abstract

Laser-plasma interactions in the novel regime of relativistically induced transparency (RIT) have been harnessed to generate intense ion beams efficiently with average energies exceeding 10 MeV/nucleon (>100 MeV for protons) at “table-top” scales in experiments at the LANL Trident Laser. By further optimization of the laser and target, the RIT regime has been extended into a self-organized plasma mode. This mode yields an ion beam with much narrower energy spread while maintaining high ion energy and conversion efficiency. This mode involves self-generation of persistent high magnetic fields (∼10^4^ T, according to particle-in-cell simulations of the experiments) at the rear-side of the plasma. These magnetic fields trap the laser-heated multi-MeV electrons, which generate a high localized electrostatic field (∼0.1 T V/m). After the laser exits the plasma, this electric field acts on a highly structured ion-beam distribution in phase space to reduce the energy spread, thus separating acceleration and energy-spread reduction. Thus, ion beams with narrow energy peaks at up to 18 MeV/nucleon are generated reproducibly with high efficiency (≈5%). The experimental demonstration has been done with 0.12 PW, high-contrast, 0.6 ps Gaussian 1.053 *μ*m laser pulses irradiating planar foils up to 250 nm thick at 2–8 × 10^20^ W/cm^2^. These ion beams with co-propagating electrons have been used on Trident for uniform volumetric isochoric heating to generate and study warm-dense matter at high densities. These beam plasmas have been directed also at a thick Ta disk to generate a directed, intense point-like Bremsstrahlung source of photons peaked at ∼2 MeV and used it for point projection radiography of thick high density objects. In addition, prior work on the intense neutron beam driven by an intense deuterium beam generated in the RIT regime has been extended. Neutron spectral control by means of a flexible converter-disk design has been demonstrated, and the neutron beam has been used for point-projection imaging of thick objects. The plans and prospects for further improvements and applications are also discussed.

## I. INTRODUCTION

Research on intense ion-beam generation got a significant impetus as a result of groundbreaking results on proton acceleration^1^ obtained by irradiating ∼*μ*m-thick foils with intense (>10^20^ W/cm^2^) ∼0.5 ps laser pulses at the Nova PW laser,^2^ where ∼10% of the laser energy was transferred to the beam with proton energies up to 58 MeV. These results, along with many other confirmatory ones from other sub-ps glass lasers, were explained on the basis of the Target-Normal Sheath Acceleration (TNSA) mechanism.^3,4^ Subsequently, in short order, essential features of TNSA were validated experimentally, including ultralow emittance^5^ and the origin of the accelerated protons being the rear target surface.^6^ TNSA was extended to heavier ions by heating the laser target to eliminate the proton-rich impurities adsorbed on laser-foil targets,^7^ enabling efficient coupling of the laser energy to the bulk ion species. By using multi-layered targets, a quasi-monoenergetic C-ion beam at ∼3 MeV/nucleon was realized,^8^ albeit with low conversion efficiency. Despite the undeniable and significant progress, the headline ion-acceleration performance (ion energy and efficiency) established early on with TNSA did not advance much for about a decade. This motivated the search for alternative acceleration schemes. Such mechanisms were found in theory and simulations, but it took some time for laser technology to enable a realistic test of those.

Testing one of those promising mechanisms became possible by operating in the relativistically induced transparency (RIT) regime.^9^ RIT comes about in a laser-heated electron plasma when, because of the increased relativistic electron rest mass, the plasma frequency decreases below the laser frequency, so that there is no longer a critical surface to reflect the light. Accessing this regime with existing lasers required sub-micron-thick laser-target foils, which in turn required the development of laser-pulse cleaning^10^ to achieve unprecedentedly high levels of contrast. Specifically, the Breakout Afterburner acceleration (BOA) mechanism^11,12^ was accessed^13^ with >10^20^ W/cm^2^, 0.6 ps, 1 *μ*m, linearly polarized laser pulses at the Trident Laser Facility.^14^ Using diamond nanofoil targets (i.e., with sub-*μ*m thickness), the BOA demonstration resulted in a leap in performance: ∼5% conversion efficiency into C^6+^ ions with energy distributions extending up to 0.5–1 GeV.^15,16^ BOA was theoretically elucidated^11,12,17–19^ and experimentally characterized.^15,20–23^ Provided proper laser-pulse-cleaning technology, sufficiently high intensity, and nanofoil solid targets, BOA was readily accessible with high-energy glass lasers without additional complications such as circular polarization or ultrashort (∼10 fs) pulses. The latter was advantageous for rapid progress because existing glass sub-ps lasers could deliver higher energies on the target at a given intensity.

Motivated by applications requiring high power density such as isochoric heating^24^ and fusion fast ignition (FI),^25^ there has been significant interest in realizing intense ion beams with a narrow fractional energy spread of ∼10%. Although BOA is ideally able to produce quasi-monoenergetic beams^11,12,25^ and although it has been done for a portion of the ion spectrum (see Ref. 25, Fig. 22), it was in practice hard to do so for the bulk of the ion distribution with diamond targets, especially with laser-spot intensities and temporal pulse shapes that are Gaussian shaped. Too much variation in plasma density interacting with too much variation in laser intensity resulted in broad energy spectra, roughly Maxwellian in shape, in the most realistic simulations as well as in experiments. Based on a limited set of simulations, it seemed that ion spectral control at high efficiency with BOA would require either more clever multi-layered targets or a super-Gaussian laser spot and temporal laser pulse shaping of sub-ps pulses to the level of ∼10 fs,^25^ which is not available at present. Therefore, important ion-beam applications seemed out of reach in the near term.

In the process of testing multi-layered foils to overcome the limitations described above, a novel mode of RIT operation was found by the LANL relativistic laser-plasma research team where the plasma self organizes in a way that results in efficient ion acceleration and a narrow ion-energy distribution, although both qualities result from separate processes. This new regime does not require multi-layered targets, although it works in such targets as well. The ion-beam experimental results, modeling, and our understanding of the mechanism are presented in Sec. III. Once available, these beams have been tested for isochoric heating of solid-density samples. This application, pursued as a collaboration between LANL and the University of Texas, Austin, is discussed in Sec. IV. A LANL interdisciplinary team has recently pursued another interesting application, where the beam plasma has been directed at a 0.5 mm Ta disk. The result has been an intense Bremsstrahlung photon beam peaked at ∼2 MeV from a small point-like source. The initial characterization and its utilization in a point-projection imaging are described in Sec. V. In that Section, it is discussed also how those experiments in turn have informed our understanding of the beam plasma.

An application where significant progress could be (and was) made with BOA beams with broad energy distributions is pulsed neutron-beam generation with laser-driven deuterium (D) beams incident on a ∼cm-thick Be “converter” disk,^26,27^ primarily by D breakup/stripping nuclear reactions. Based on the higher efficiency and average ion energy of BOA D beams, the resulting multi-MeV neutron-beam yields (∼10^10^ neutrons in ∼1 sr and ∼1 ns) greatly exceed other attempts.^28–34^ Progress since that work,^26^ pursued as part of a collaboration between LANL and the Technical University of Darmstadt, is summarized in Sec. VI.

The laser parameters and key diagnostics are discussed in Sec. II. To provide a perspective for the future, our present understanding of how to optimize laser parameters for ion acceleration in this regime is presented in Sec. VII. A summary of this work is presented in Sec. VIII.

In addition to the work described herein, we acknowledge the research by other groups that have considered and are experimenting with relativistic laser-plasmas in the RIT regime.^35–45^

## II. EXPERIMENTAL SETUP

### A. Trident laser facility

All the experiments discussed herein have been carried out at the LANL Trident laser facility.^14^ These experiments involve the interaction of an intense laser pulse with a solid-density nanofoil. The laser pulse has a wavelength of λ_L_ = 1.053 *μ*m. For these experiments, the pulse duration was kept in the range of 0.60–0.65 ps FWHM, and the laser-energies were varied within the range of *E*_L_ = 60–90 J, with 80 J being typical. The laser is focused on the target to a nearly diffraction-limited spot with a peak intensity *I*_L_ of 2 × 10^20^ W/cm^2^ by a 9-in. f/3 optic or 8 × 10^20^ W/cm^2^ with f/1.5. Two key features of the Trident laser are an ultrahigh contrast ratio^10^ and the elimination of all pre-pulses. These features enable the use of nanofoil solid targets to reach the RIT regime. The details of the remaining “cleaned” pulse still have an important effect in the “pre-plasma” generated by the non-ideal, premature rise of the laser intensity that determines whether this new mode of operation can be reached. (The risetime of the ps-scale pre-pulse is also an important factor in the evolution of laser plasmas made with microfoil targets.^46^)

The reflected laser light is used as a diagnostic of RIT. It is detected with a single-shot Frequency Resolved Optical Gating (FROG) diagnostic with a ∼50 fs time resolution, as described in Ref. 47. The disappearance of the reflected pulse indicates the onset of RIT. Moreover, importantly for our purposes, it also provides the time-resolved critical-surface motion until RIT ensues. A proper time history of this motion has proven to be an essential signature of high quality shots. Once the correspondence with the full FROG data are understood, the measured time-integrated spectrum can be used for an immediate evaluation of the shot without having to run the full FROG analysis.

**FIG. 1.**
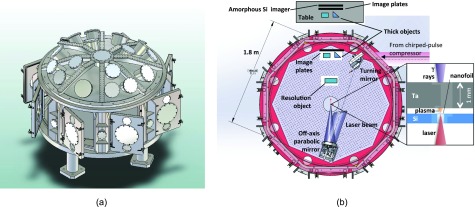
(a) Isometric projection of the Trident West target chamber in its baseline configuration. (b) Top view of the experimental configuration used for the γ-ray generation experiments. The “kaleidoscope” object for point-projection high-resolution imaging and the thick objects for near-contact imaging are not all used simultaneously. The inset shows a top-view detail of the laser-target nanofoil and Ta-converter arrangement. The nanofoil-converter registration is done by spacers off the field of view. Magnets and shielding to avoid direct electron and scattered γ-ray detector irradiation are not shown.

The experiments on ion-acceleration (Sec. III) and on isochoric heating (Sec. IV) and the earlier portion of those on neutrons (Sec. V) were fielded in the North target chamber (NTC), described in Fig. 4 in Ref. 14, supplementary Fig. 4 in Ref. 48, and Fig. 1 in Ref. 26. Those publications include overhead chamber schematics that show the relevant diagnostic layouts. The experiments on gamma-ray generation (Sec. V) and neutron-imaging (Sec. VI) were fielded in the West Target Chamber (WTC),^49^ as shown in Fig. 1(a). It is made out of 5052 aluminum, with a 1.5 in. (3.8 cm) wall thickness. It encloses an optical table on the equatorial plane. The table and chamber are octagonal in shape at the equator, with a radius of 1 m. The chamber has eight flat sides 36 in. high, with large square holes covered by flat aluminium doors 2.59 in. thick, and some of them were modified to incorporate distinct ports per laser-beam and diagnostic-access requirements. These doors allowed the placement within the chamber of large heavy metal pieces for x-ray shielding and large-area image plate (IP) detectors. Figure 1(b) shows a representative overhead chamber schematic of the diagnostic layout used on the experiments on gamma-ray generation. As shown in the figure, X-ray and gamma-ray detection was done both inside the chamber near the outer radius and outside through the Al-walls. In the alternative WTC configuration where the ion-beam spectra were measured, our high-resolution Thomson-parabola (TP) ion spectrometers (see Sec. II B 1) were deployed in place of the diagnostic table shown on the top of Fig. 1(b), with their 300 *μ*m pinhole aperture located at 1.3 m from the target-chamber center (TCC). The arrangement of the TPs in the WTC is otherwise the same as shown for the NTC in Fig. 4(b) in the supplementary section of Ref. 48, with the same observation angles relative to the laser-propagation direction, 0° and 11°.

### B. Key diagnostics

#### 1. Ions

All of the spectral measurements of ion energy (*E*_i_) herein have been made with two newly developed high-dispersion Thomson parabola spectrometers (Ref. 48, Supplementary Fig. 4). They feature longer electrodes (up to 50 cm), shorter magnetized length (0.82 T over 10-cm long) and longer drift length relative to our prior ones. This has enabled better separation of traces from different charge states. The two TPs were located side-by-side, on-axis and 11° off-axis, enabling simultaneous data collection. The detector is a rectangular piece of the Fujifilm BAS-TR image plate (IP), scanned with a Fujifilm Model FLA-7000 scanner, cross-calibrated against CR-39 nuclear track detectors.

#### 2. Neutrons

Neutron flux at selected locations relative to the laser-propagation directions are measured using standard neutron bubble-detectors by Bubble Technologies. These detectors are sensitive to neutrons above ∼100 keV and provide a robust yield measurement, especially when corrected for the modest spectral sensitivity variation. The yield has been cross checked with a Rhodium-activation neutron detector.

Following the initial experiments presented in Ref. 26, the neutron-time-of-flight (nTOF) diagnostic has been upgraded to determine the neutron-energy spectrum accurately under the challenging conditions of our experiment. Specifically, we have utilized a new generation of metal-channel photomultiplier tube (PMT) with a planar photoelectron collection structure developed for nuclear-safeguard work at LANL.^50^ This PMT maintains a linear response over a wider dynamic range, important in this high pulsed-flux environment.

For neutron imaging, we have utilized a computer-driven Perkin-Elmer 40 cm × 40 cm amorphous-Si (a-Si) flat panel display with a 2.4 mm thick ZnS(Cu)-in-plastic screen made by RC Tritec Ltd. This detector has a ∼200 *μ*m pixel size. This robust setup is used in other experiments at LANL as well.

#### 3. Gamma radiation

Gamma-ray images have been recorded in our experiments using two types of detectors. One is image plates (IPs), which we have calibrated independently, as described below. The pixel resolution is chosen at the time of IP scanning, typically 100 *μ*m. The other one is the computer-controlled PE amorphous Si flat-panel display (150 *μ*m pixel size) optimized for photon detection with a 0.38 mm thick Ta-foil intensifier and gadolinium oxysulphide (LANEX) scintillation screen.

The determination of gamma-ray dose depends on the IP calibration at high-photon energies. There is not much work published in this area,^51,52^ and the publications disagree significantly. Therefore, we were motivated to do our own, presented here. We utilized several calibrated radioactive sources used in LANL Nuclear Safeguards research, purchased from Eckert and Ziegler from their Reference, Point Source product line. Based on their certificate of calibration, we calculated the activity for the day that the measurement was done. The sources that were used are listed in Table I.

**TABLE I.** Radioactive sources used in our photon-sensitivity calibration of IP type SR. The averages, when used (in bold), are weighted by the emission probability and the transmission through the Ta filter used (see Table II).

Source	Energy (MeV) of relevant γ rays	γ/Disinteg.	Source	Active area Dia. (mm)	Activity (*μ*Ci)
^133^Ba	0.276	0.071	Type D	5	106
	0.303	0.183			
	0.356	0.620			
	0.384	0.089			
	**Wt. Avg. 0.349**	**0.963**			
^22^Na	0.511	1.807	Type D	5	10.2
	1.257	0.999			
^137^Cs	0.662	0.850	Disk type	1	14.5
^60^Co	1.173	1.000	Type D	5	93.4
	1.333	1.000			
	**Wt. Avg. 1.253**	**2.000**			
^228^Th	0.583	0.304	Type D	5	11.5
	0.911	0.258			
	2.614	0.356			

**TABLE II.** Source-emission measurements recorded in BAS-SR 2040 IP.

Source	Ta filter thickness (cm)	Exposure time (s)	Distance, IP-active layer (cm)	Measured net PSL
^133^Ba	0.28	300	2.158	0.20 ± 0.01
^22^Na	0.10	300	0.548	1.10 ± 0.10
^137^Cs	0.15	360	0.390	1.21 ± 0.12
^60^Co	0.05	300	1.928	0.56 ± 0.04
^228^Th	0.30	300	0.658	0.20 ± 0.04

In such calibrations, it is important to utilize proper filtering to exclude beta and low-energy photon peaks, to which the IP is much more sensitive than it is to the gamma rays of interest. Given the modest activity of the sources we have used, a small separation between the source and the IP has been necessary. Therefore, it is critical to determine accurately the internal geometry of the source, specifically the location of the active layer. It is also important to account for the finite diameter of the active area. Table II shows the measurement particulars with each of the sources.

**FIG. 2.**
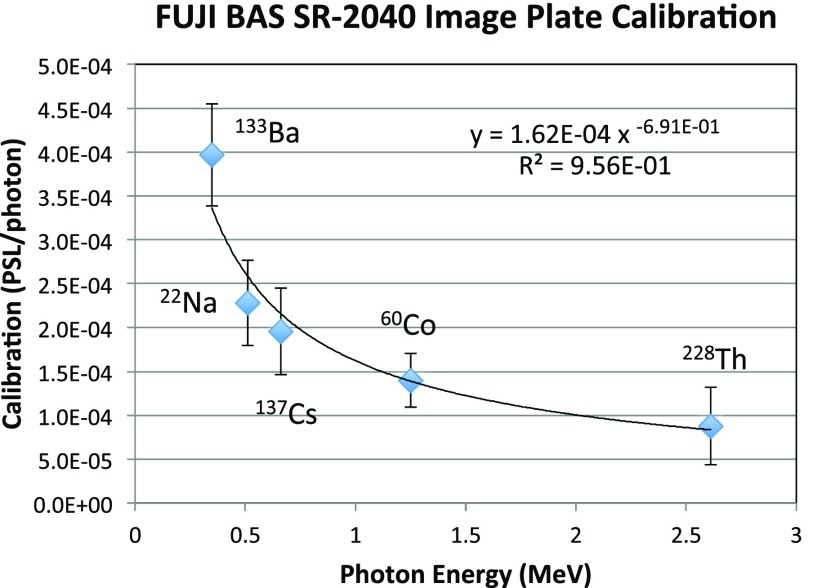
Measured calibration of IP type SR.

Our calibration results are shown in Fig. 2. Our measured sensitivities lie much above the values in Ref. 51 and somewhat above the values presented in Ref. 52, although our scatter off a monotonic functional fit is much less. Therefore, our dose determinations are conservative relative to other published calibrations. Although they can vary from case to case, the average contributions to our error bars can be listed in quantitative order as uncertainties in source-IP distance, the accuracy of other calibrations (with Na22 and Th228 sources), the net PSL value, latency correction, source activity, exposure time, and the diameter of the active area.

#### 4. Plasma expansion

In these experiments, the materials isochorically heated by the laser-driven ion beam are typically foils with thicknesses ∼10 *μ*m. The heating magnitude and the consistency with equations of state tabulated in the SESAME database^53^ have been tested by observing the free expansion of the plasma into vacuum.^54^ In these experiments, we have taken advantage of the fact that, although the plasma expands adiabatically and cools, the expansion speed of the plasma leading edge is constant and reflects the initial temperature,^55^ which is the datum of interest. So, we can measure the expansion over a time longer than the plasma-cooling time to gain accuracy. Moreover, because of the volumetric uniformity of the heating within the sample, we are confident that our plasma expansion measurement reflects the bulk sample temperature.^56^

The expansion of the plasma leading edge has been diagnosed by visible shadowgraphy. Specifically, the interface is backlit with a 660-nm probe-laser beam. The transmitted light, with appropriate attenuation, is imaged into the entrance slit of a Hamamatsu 4187 streak camera. The interface was clearly visible as a shadow (a step in illumination) with sub-*μ*m resolution. The image is streaked in time, with a total window of ∼10 ns in the photocathode. A train of sub-ns optical pulses are also delivered to the slit by an optical fiber, which serves to verify and cross calibrate the time base of the streak camera. This system has ample resolution to resolve the measured expansion velocities, in the 5–10 *μ*m/ns range, resulting from bulk heating to the few eV range.

In a recent experimental block for this project, a time-resolved optical pyrometry diagnostic to measure the temperatures of these ion-heated plasmas was fielded by our colleagues from the University of Texas at Austin.^57^ In this diagnostic, the back surface of the ion-heated sample was imaged to the slit of a Hamamatsu C-7700 streak camera, showing the spatial extent and temporal progression of heating by the ions. With a temporal resolution of <5 ps, we were able to verify that the heating time was consistent with an ion source that is quasi-monoenergetic at several MeV/nucleon. The measured surface temperatures were consistent with the high ion fluence, heating to >1 eV with the ion source separated from the sample by a gap greater than 1 mm. Furthermore, the spatial resolution of the diagnostic showed that the angular extent of the ion beam is quite small, approximately 11° FWHM.

## III. LASER-DRIVEN INTENSE ION-BEAMS WITH NARROW ENERGY SPREADS

### A. Background

In all the experiments discussed in this article, the laser-plasmas reach RIT typically near the peak of the laser pulse. This condition, found to be optimal, is achieved by matching the nanofoil target thickness (for the given areal density) vis-à-vis the peak laser intensity and pulse length. The onset of RIT, when the full electron population across the plasma thickness has been heated to relativistic temperatures and the relativistic plasma frequency drops below the laser frequency, depends on the electron heating rate and on the secular density decrease due to the hot-plasma disassembly. To optimize the laser-plasma coupling, the target thickness must be adjusted to avoid either premature disassembly relative to the peak of the pulse or too high an areal density for the heating rate that the laser can supply. The finite pre-pulse increases the optimal thickness. For existing high-power, high-intensity lasers, the optimal solid target thickness lies in the sub-*μ*m range. An experimental demonstration of thickness optimization can be found in Fig. 3 of Ref. 20.

There have been two technological keys to enable access to the RIT regime. The first is the ability to fabricate and properly mount nanofoils in an economical fashion (see for example Ref. 58). The second is to clean the laser pulse to a sufficient extent that its early rise or pre-pulses do not destroy the target prior to the arrival of the high-intensity portion of the pulse. This has been done by cleaning the pulse itself,^10,59,60^ and in other facilities, by using plasma mirrors.^40^

Even in the regime of broad ion-energy distributions, we have found in our simulations with the Vector-Particle-In-Cell (VPIC) code^61^ that the choice of initial plasma conditions and resolution (spatial and temporal) affects the early plasma evolution and expansion and therefore the onset of relativistic transparency. This has a significant impact on other parameters, including ion-acceleration performance. The optimal balance of computational efficiency versus fidelity to ensure convergence of the simulation results is an active field of study. More generally, while the simulations and interpretation presented in Sec. III C embody our best present understanding, modeling work on solid density RIT plasmas continues in order to challenge and improve our understanding. Such work includes comparisons of selected 3D versus 2D simulations of either polarization.^62^

Because of the dependence of quantitative modeling predictions on the chosen initial conditions, we feel compelled to use a reflected-light FROG diagnostic in our experiments in order to ascertain truly the regime of the laser plasma interaction and to ensure consistency of operation. This diagnostic imperative is even more critical when operating in the enhanced mode discussed here. We have found, in part by accident when the laser pulse shape has been spoiled inadvertently by leakage from the long-pulse beam in multi-beam experiments, that a ∼ns laser-pulse pedestal/pulse peak ratio as small as ∼10^−12^ can spoil this enhanced mode. Although we have derived much understanding from modeling accurately the pre-plasma expansion from the ∼ns laser pulse pedestal with a radiation hydrodynamics code, there is hardly a quantitative predictive capability in place anywhere for this purpose. It is the use of the reflected-light FROG that has enabled us to benchmark our modeling and to ascertain the correlation of the crucial pre-plasma dynamics with ion-beam performance. This allows an immediate determination of whether the desired regime has been reached in any given shot, independent of ion-beam measurements.

Initial experiments on Trident in the RIT regime^13,15^ utilized diamond-like nanofoils developed collaboratively by scientists from Ludwig Maximilians University in Munich, Max Planck Quantum Optics and Maier-Leibnitz Inst. in Garching, Kurchatov Institute in Moscow, and Los Alamos.^58^ These nanofoils delivered superior performance in C-ion acceleration in the RIT regime relative to thicker (>750 nm) targets where TNSA dominates.^21^ (Presently, diamond nanofoils suitable for laser targets are available commercially from companies such as Applied Diamonds in Delaware, USA.) Yet, even at optimal diamond thickness (∼200 nm for f/3 laser focusing) with nominal full energy pulses (∼80 J), the observed spectra were broad. This is consistent with VPIC simulations of those experiments.^18^ Specifically, those simulations never exhibited strong, self-generated magnetic fields (*B*) that could impact the laser-plasma evolution. As would become evident in later experiments, these targets were affected adversely by the small but finite laser-pulse contrast on Trident.

### B. Experimental results

The utilization of Al nanofoil laser targets resulted in the discovery of the enhanced self-organization mode. These foils delivered narrow ion-energy peaks with relatively high laser-ion conversion efficiency and a smaller beam divergence (up to 17° HWHM).^48^ The dominant charge state was Al^11+^ along with the ubiquitous proton beam from target-surface impurities and a small Al^12+^component. As indicated by an atomic ionization calculation of aluminum for Trident laser parameters (*f/*3 focus), the preponderance of Al^11+^ is due to the huge inner-shell gap between the Al^11+^ and Al^12+^ ionization potentials. In this self-organization mode, the average energy of the Al^11+^ ion distribution measured above the instrumental cutoff is only slightly lower than the spectral peak value, and the average *E*_i_/nucleon remains as high as what was observed before.^20^ For example, the average of the quasi-Maxwellian C^6+^ energy distribution from ∼100–200 nm-thick diamond nanofoils illuminated by 80 J focused to *f/*3 is 67 MeV (5.7 MeV/nucleon or MeV/u),^20^ while a typical shot with 110 nm Al foil yields a spectral peak at 165 MeV (6.1 MeV/u) and an average energy of 130 MeV.^48^ (This ion beam is used in the isochoric-heating experiments discussed in Sec. IV.) A summary of the performance achieved in this enhanced regime is presented in Table III. In all cases, according to measurement and simulation, RIT is reached close to the peak of the laser pulse.

**TABLE III.** Laser-driven ion-beam performance with optimal target thickness.

	Laser target	Focus optic	Intens. (10^20^ W/cm^2^)	Ion spectral peak		En. spread (FWHM)	Effic. (%)
				MeV	MeV/u		
1	Al 110 nm	*f/*3	2	165 Al^11+^	6.1	7	5
2	Al 250 nm	*f/*1.5	8	310 Al^11+^	11.5	41	4
3	110 nm Al/10 nm C	*f/*3	2	80 C^6+^	6.7	15	…
4	250 nm Al/10 nm C	*f/*1.5	8	120 C^6+^	10.0	54	…
5	C 110 nm	*f/*3	2	None	None	…	…
6	C 250 nm	*f/*1.5	6	220 C^6+^	18.3	23	4
7	C 250 nm	*f/*1.5	8	None	None	…	…

Besides pure Al in rows 1 and 2, Table III summarizes two other important cases which provide important insights into this regime. Rows 3 and 4 show the results for multi-layer Al/diamond foils. The Al layer, which constitutes most of the areal density of the target, is placed facing the incoming laser. Therefore, the pre-plasma and subsequent target disassembly dynamics are expected to be similar to the pure Al-foil case. However, the diamond layer on the rear side provides the bulk of the observed beam ions. C^6+^ is accelerated to a similar *E*_i_/nucleon as the Al^11+^ ions in the pure foil, consistent with their similar charge/mass ratios. In other words, the favorable dynamics of solid Al can be harnessed to make a beam of the ion species of our choice.

Another important case, diamond foil laser targets, is summarized in rows 5–7. Consistent with prior findings, in the case of a full-energy Trident pulse illuminating a diamond nanofoil of optimum thickness, a strong spectral peak is not observed, and the pre-plasma dynamics (the expansion velocity of the critical density in time) are qualitatively different from those in the enhanced (spectral peak) case. Yet, by backing off in laser energy (row 6), similar pre-plasma dynamics and a spectral peak are recovered, to yield in fact our highest *E*_i_/nucleon, although not the sharpest spectral peak. We attribute the transition to the fact that by lowering the laser energy, the pedestal energy is also lowered.

### C. Simulations

To elucidate the difference between Al and diamond target performance and the mode transition seen with diamond, we turned to 2D radiation-hydrodynamic simulations with the code HYDRA, tuned to match the observed target expansion velocities.^48^ These simulations indicate that with the same level of pre-pulse, the diamond target disassembles faster than Al during the ns laser pedestal, resulting in a much lower peak plasma density in the C-plasma than in the Al-plasma when the main laser-pulse arrives. This has a profound effect in the plasma evolution, specifically a much lower self-generated magnetic field and no discernible self-organization. The laser pedestal decrease of 25% corresponding to a 60 J shot (rather than 80 J) is seen in the combined hydrodynamics and VPIC simulations to arrest the expansion and recover the enhanced dynamics, leading to the spectral peak.

The laser-pedestal interaction with the target plasma was also run in 1D with the code HELIOS^63^ to generate plasma profiles to serve as initial conditions for the VPIC simulation of the relativistic laser plasma. These 2D simulations, described in detail in Ref. 48, have been invaluable in understanding the nature of this enhanced regime. The problem required a series of exceedingly large-scale simulations over a range of peak electron densities (*n*_e_ = 125–250 *n*_cr_), charge/mass heavy ion states (0.41–0.5), initial electron temperatures (1 keV–32 keV), peak laser intensities, and laser linear polarizations (p and s). The Debye length was resolved from the beginning of the simulation. In order to capture the full dynamics and to avoid the reflection of electrons back into the plasma from the simulation boundary that is charged up by escaped electrons, the simulation box had to be made very large, over 200 *μ*m along the axial dimension and 50 *μ*m transversely. The simulation had to be run until 2.20 ps, well after the laser leaves the simulation box.

**FIG. 3.**
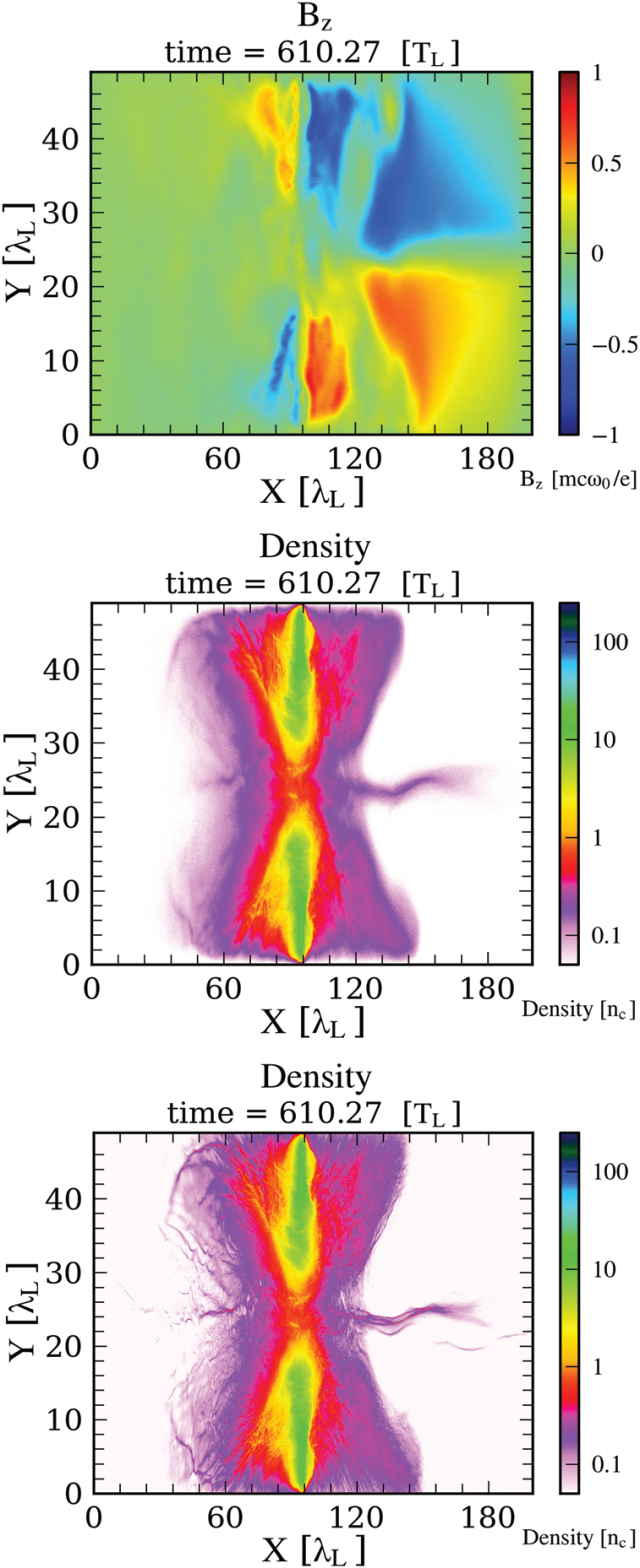
Profiles at 2013 fs (610 optical cycles). Laser propagation is from left to right. The initial target location is *x* = 95 λ_L_. Top: Magnetic field profile. The range is ±10 kT. Middle: Electron-density profile, illustrating the plasma jet along the central channel. Bottom: Ion density distribution. The ion energy distribution has not yet narrowed.

**FIG. 4.**
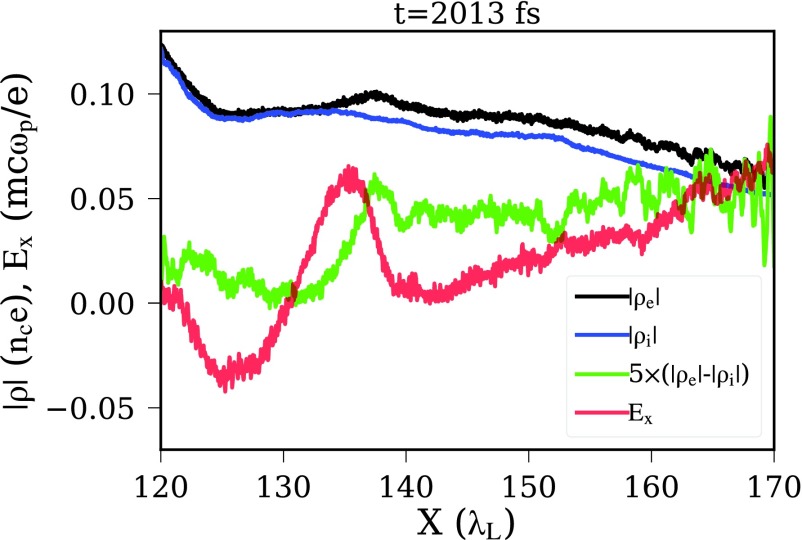
Channel-averaged profiles of electron and ion density, net charge, and the resulting longitudinal electric field.

There are a few salient results from our VPIC simulations of these laser-plasmas concerning the nature of this enhanced mode versus the one previously documented for BOA ion acceleration:

(1) When the plasma disassembly induced by the non-ideal early portion of the laser pulse is not controlled, i.e., when the maximum electron density across the axial plasma cross section falls significantly below ∼250 *n*_cr_ at the onset of RIT, the self-generated *B*-field is too weak, the hot electrons are not strongly magnetized, and the plasma evolution is as reported before. No discernible self-organization nor ion spectral peaking is observed experimentally in otherwise identical simulations as those where the enhancement is seen.
(2) On the other hand, in the optimized case where the electron density is sufficiently high at the peak of the pulse, the dynamic is different. Before transparency, strong azimuthal *B*-fields (>10 kT) are present and are confined, as expected, to the front and rear plasma surfaces (see Movie in Ref. 48). Shortly after transparency, forward-moving hot electrons, influenced by the B-field, break out massively from the rear surface as a jet, and that current supports a fast expansion of the azimuthal *B*-field at the rear surface of the plasma into space, to form a toroidal structure of the azimuthal B-field around the laser-propagation axis. The plasma settles into a characteristic state, where the electron current is pinched on axis to keep driving the self-consistent azimuthal *B* field. The channel does not stay completely straight—it actually kinks a bit. The ions, although they are weakly magnetized, are constrained to overlap the electrons to preserve gross quasineutrality. The magnitude of *B* vanishes on the axial channel, but it increases sharply at the edge of the channel, and any electron that veers off the channel is strongly magnetized. Figure 3 shows selected profiles from the VPIC simulation of the Z_eff_/A = 0.5 (C^6+^) plasma driven with the f/1.5 beam at moderate intensity, the case in Table III, row 6 that yields a peaked ion-energy spectrum. Notice the kink at *x* ≈ 140 λ_L_, where the ion energy distribution becomes peaked at a later time.
(3) Since most electrons in the channel do not have a nearly zero pitch angle, and the channel has small kinks, most electrons cannot traverse along the channel without being repeatedly trapped by the magnetic field near the edge, de-trapped, and, upon crossing the axis, trapped again at the other side of the channel. Therefore, although individual electrons may be moving or gyrating at high energy (at nearly the speed of light *c*), the flow of the electron population along the channel is impeded. As a result, sharp and localized electron-density variations that are not fully neutralized by the much slower ions can develop transiently, and strong, localized, transient longitudinal electric fields can appear. Selective tracking of electrons of the subpopulation ultimately associated with the ion spectral peak have greatly clarified these dynamics. To illustrate the point, Fig. 4 shows the profiles (averaged over the channel width) of electron and ion charge density, the net charge density, and the resulting longitudinal electric field. The localized positive electric field appearing at the kink in the channel persists for ≈180 fs, playing a critical role in the narrowing of the ion energy distribution.
(4) By the time the laser leaves the plasma at ≈1820 fs, the ion population corresponding to the beam is also arranged as a thin jet along the channel, roughly overlapping the electron jet (Fig. 3, Bottom). At this point, the ion-beam is not quasi-monoenergetic, but it is chirped, i.e., the leading edge is fastest, and so, the ion energy decreases monotonically towards the tail end, which moves slowest. In other words, the ion jet is moving and expanding longitudinally.
(5) The longitudinal electric field in Fig. 4 overlaps a portion of the ion filament along the channel. The ions at that location are accelerated and catch up with the ions moving ahead, which had acquired higher energy during the laser-plasma interaction. Thus, the energy distribution is modified, or in beam-physics parlance, part of the ion distribution is rotated in longitudinal phase space.
(6) By time 2030 fs, the longitudinal electric field turns off, and the distribution has become peaked.

**FIG. 5.**
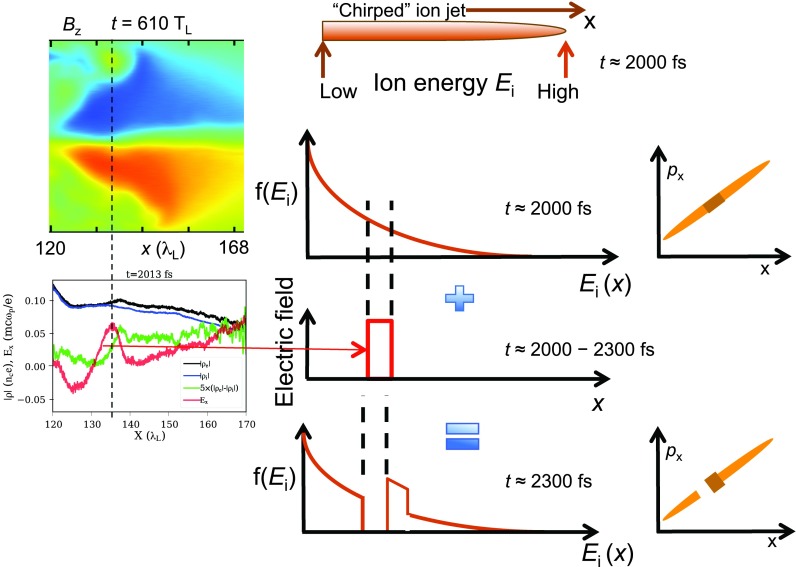
Self-organization process leading to peaking of the ion-energy distribution.

The process is summarized graphically in Fig. 5. After the laser is gone, at time approximately 2000 fs in the simulation, the laser-plasma interaction has produced an ion plasma that lies along the channel and is chirped in energy, as illustrated in Fig. 5 (top center). The magnetic field (top-left) has impeded the forward-transport of electrons, leading to a longitudinal electric field that is localized in space and in time (Fig. 5, middle), lasting for the period of ≈2000–2300 fs. This field accelerates a spatial portion of the ion plasma, which corresponds to an energy slice, because of the chirp. At ≈2300 fs (Fig. 5, bottom), the affected portion of the ion distribution has been accelerated to match the energy of the ions moving ahead, thus peaking the distribution.

Additional experiments to detect these large magnetic fields directly have been performed, and modeling is underway to interpret the results. This work will be presented in a future journal article. Two additional important questions remaining for further work are how to further optimize and control this self-organization process and to what extent externally imposed fields (e.g., with laser-driven miniature coils) or an externally injected electron bunch may be useful to manipulate the beam phase space.

## IV. ISOCHORIC HEATING OF DENSE PLASMAS USING INTENSE ION BEAMS

Laser-driven ion beams with a narrow energy spread are ideal for isochoric heating of macroscopic amounts of dense matter. Since these beams are neutralized and they are born in ps timescales, they are immune to the conventional space-charge and current limits of conventional beams and thus are able in principle to deliver a significantly higher power density per Joule, from a device that is more compact than a conventional accelerator. The ions lose energy mainly by coupling to electrons. In practice, because of the finite energy spread that expands the beam longitudinally as it flies, it is hard to deliver the ion pulse in a time shorter than ∼10 ps, which is similar to the electron-ion temperature equilibration time. Therefore, laser-driven ion beams are an appropriate tool for near-equilibrium heating, albeit fast compared with hydrodynamic timescales. If non-equilibrium electron heating is needed (∼ps and below), there can be no significant difference in time of flight, which leaves x-ray free electron lasers (XFEL) as the option of choice at present. The XFEL at LCLS has demonstrated the ability for bulk isochoric heating of thin, low-Z samples,^64^ emerging as a viable and practical alternative in cases where the heated-sample volume can be small.

There are two types of isochoric heating applications, and both benefit strongly from a narrow ion-energy spread. The first type is where maximum heating is desired and the ion beam ranges within the target, such as ion-driven fast ignition (FI).^25^ In FI, a minimum ion energy is needed to penetrate through the coronal plasma to the compressed D-T fuel (*E*_i_ ∼400 MeV for C ions and ∼10 MeV for protons). For ion FI with a standoff ∼1 cm between the ion-beam laser target and the capsule, the maximum energy spread that can be tolerated is ∼10%^65^ in order to deliver the power density to the compressed fuel required to ignite. The work discussed in Sec. III represents a significant boost for the prospects of ion-based FI with heavier ions. Specifically, the required energy spread has been demonstrated at an acceptable laser-ion conversion efficiency, and an ion energy for C ions (*E*_i_ = 220 MeV) just over half of the FI requirement (*E*_i_ = 400 MeV) has been demonstrated at an intensity of 6 × 10^20^ W/cm^2^ with the conventional laser technology available at Trident. With an intensity scaling in the RIT regime of *E*_i_ ∼ *I*_L_^1/2^, 400 MeV would require 2.4 × 10^21^ W/cm^2^, accessible with existing glass laser technology.

The second type of emerging application is uniform volumetric heating of a macroscopic sample, as demonstrated recently on Trident using the Al beams listed in Table III, row 1.^54,56^ Examples of experiments enabled by this technique include the equation of state (EOS) studies of warm-dense matter, shock-physics experiments with arbitrary phase-space paths (off the principal Hugoniot or the isentrope from room temperature), and hydrodynamics experiments with arbitrary initial conditions. For uniform heating, the ion energy and the sample thickness and material are chosen to ensure that the beam does not range out in the material, exploiting the relatively uniform spatial loss rate of the ion energy far from the Bragg peak. In the case described in Ref. 54, an area of order (0.5 mm)^2^ on solid foils of thickness ∼10 *μ*m was heated within 25 ps to a few eV, and the theoretical equations of state of diamond and Au were tested in that regime.

With this technique, a low ion-energy spread is necessary for two main reasons. The first one is that the ion pulse must be significantly shorter than hydrodynamic disassembly time of the sample, which scales with electron temperature as *T*_e_^−1/2^. The second is that, given the need to avoid ranging out within the sample, a broad energy distribution is at best wasteful of energy and at worst fatal, as in the case of a Maxwellian energy distribution. In that case, the front face of the sample is heated preferentially by the low energy portion of the spectrum, spoiling the heating uniformity along the cross section of the sample. Perhaps counter-intuitively given the high electron-ion collision rate, heat conduction is much too slow (by orders of magnitude) to equalize the temperature following such non-uniform heating.

Another interesting point is that, although a mono-energetic ion beam is good for this application, it is not optimal. Based on calculations with the SRIM^66^ ion-energy deposition code, we were surprised to learn that the Al-ion beam on Trident with a finite energy spread produced slightly more uniform heating than a hypothetical mono-energetic beam at the same average energy.^54^ This illustrates the need for careful modeling in such experiments, especially if precisely tuned conditions for highly accurate measurements are required.

## V. GAMMA-RAY GENERATION WITH RELATIVISTIC TRANSPARENT PLASMAS

Although our focus has been on ion acceleration, we have also explored the possible utility of the beam plasmas discussed above for generating high-energy photons (>100 keV), hereafter called simply gamma rays. These exploratory experiments have utilized the Trident beam focused with the f/3 optic on Al nanofoils (Table III, row 1) and other nanofoil targets such as Al/diamond and Ti. While f/3 does not yield the hottest electrons, it has been used for operational convenience in this exploratory phase of the work. We are interested in measuring the Bremsstrahlung gamma rays generated by the interaction of the beam-plasma electrons with a high-Z material. Besides the possibility of a unique source with a very small source size and high dose per shot, we are interested in what these measurements can tell us about the beam plasma.

We have placed a thick disk (0.5–1 mm) of Ta (the converter) parallel to the laser target, located 100–400 *μ*m beyond it. This arrangement is similar to the one in an earlier study on the production of Kα x-rays.^67^ Here, the distance is chosen to be far enough to avoid interfering with the plasma dynamics at the rear of the laser target but close enough to capture the beam plasma before it undergoes significant expansion. As far as a gamma-ray source is concerned, there are two considerations of interest. Although the beam-plasma electrons co-move with the ions and therefore have a relatively slow net velocity (∼0.1c), their temperature (from ponderomotive laser heating) is high (multi-MeV). Therefore, upon arrival at the converter, the beam electrons may execute multiple passes and convert efficiently (as with a thick target) while maintaining a small source size, suitable for point-projection imaging. Although these laser targets become relativistically transparent, we can rule out direct illumination of the converter as the source of the gamma rays. Most of the laser energy is in fact absorbed by the target plasma, and the transmitted beam (∼5%–10% of the energy) would be significantly out of focus if it reached the converter. If we were making a significant dose of gamma rays by direct illumination of the converter, likely we would see a double source when imaging.

### A. Point-projection imaging

**FIG. 6.**
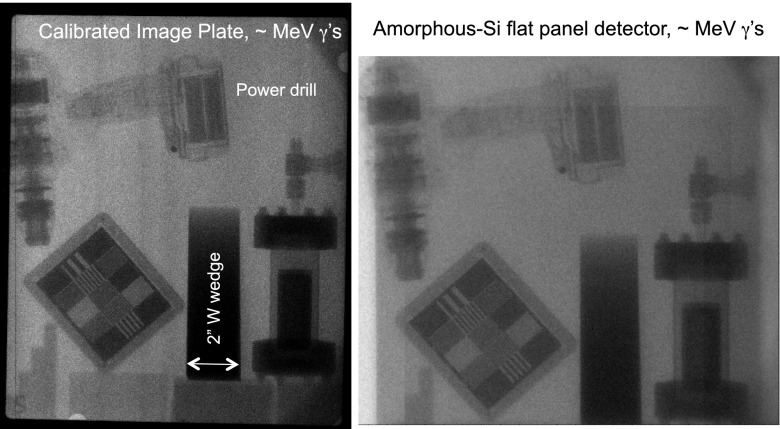
Point projection radiographs of thick objects recorded on collocated image plates (left) and on an a-Si gamma-ray detector (right).

This source has been used for point-projection imaging of thick objects on the amorphous-Si gamma-ray detector described in Sec. II B 3, as well as on image plates. The configuration for these experiments is shown in Fig. 1(b). Figure 6 shows those radiographs for a shot using an Al nanofoil and a 0.5 mm Ta converter placed ≈200 *μ*m beyond the target. The W wedge is 2 in. wide by 7 in. long by 2 in. maximum depth. The magnet for the iWASP spectrometer^68,69^ (∼0.5 T) was placed after the converter to deflect any electrons that may possibly impact the detectors or provide an uncontrolled source of x-rays. For Fig. 6, the detectors were placed 1.45 m away from the source at the West TCC. The IP was placed in front of the Si detector, a 0.46 mm thick Ta sheet was placed in front of the IP, and the objects were placed in front of that. The detectors viewed the source through the 2.59 in. Al door, which only transmits 5% of photons at 0.1 MeV and 0.15% at 50 keV. In addition, the Ta sheet transmits only 0.3% of 0.2 MeV photons and 0% at 0.1 MeV, making us confident that these images were made by gamma rays.

### B. Gamma-ray source size

**FIG. 7.**
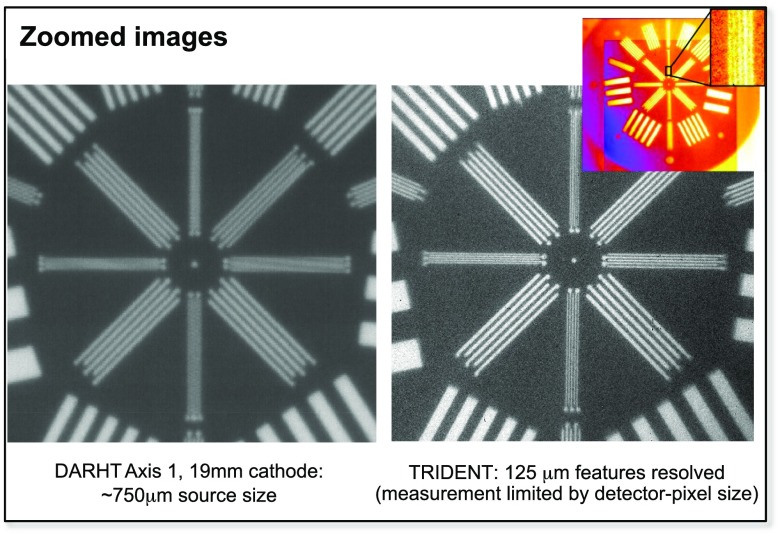
Gamma-ray point-projection radiographs of the AWE Kaleidoscope object, taken at DARHT (left) and at Trident (right).

In order to estimate the source size, the “kaleidoscope” resolution calibration object originally designed by the Atomic Weapons Establishment (AWE) in the UK was used. It was placed along the line of sight inside the target chamber to realize a 3.8× magnification. This object is an excellent reference that has been used in the LANL Dual Axis Radiographic Hydrodynamic Test (DARHT) Facility accelerator,^70^ as well as in other experiments on laser-driven x-ray sources. It is basically a Tungsten disk 1.6 cm thick with several cutouts of parallel-line patterns of varying widths and separations. The radiographs of the kaleidoscope taken at DARHT axis 1 with the 19 mm cathode and at Trident are compared in Fig. 7. The gamma-ray-spot size at DARHT corresponds to a relatively low x-ray dose, while a full dose shot (1.7 kA beam) has a 2 mm time integrated spot size (variable in time).^71^ On Trident, the smallest feature (125 *μ*m) is resolved, indicating a source size of that order. Unfortunately, the pixel size of the Si-detector is also similar in size, and so, further measurements are necessary to ascertain the source size more precisely. Nevertheless, based on our work and that of others, it appears that a point-like, directed gamma-ray source driven by intense high-energy lasers can be made that is >5× smaller than the present generation of intense sources based on conventional electron accelerators.

### C. Gamma-ray beam divergence

The gamma-ray beam has a typical estimated FWHM solid angle of ≈0.07 sr (≈8° half angle). The forward-directed Bremsstrahlung cone from relativistic electrons on a thick target^72^ has an angular spread of the same order as the angle we measure. Therefore, the beam-plasma hot electron distribution cannot be isotropic, but rather it must be nearly one dimensional along the propagation direction. This is perhaps not surprising given that only electrons with a very small pitch angle can escape along the channel in the magnetic field at the rear of the laser plasma. We also observe that the gamma-ray beam direction can vary from shot to shot by ∼5° off the laser-propagation direction.

**FIG. 8.**
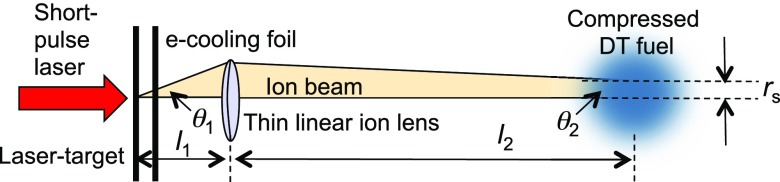
Laser-driven ion-beam focusing geometry for fast ignition.

This observation is very good news for the prospects of focusing tightly the ion-beam, which would be much worse if the electron distribution turned out to be isotropic. It means that the angular spread of the beam at the beginning of its flight is small. If soon after its birth the beam-plasma hot electron population is charge-exchanged (i.e., the beam plasma is cooled) as described in Refs. 73 and 74 by a microfoil placed behind the target at a similar separation as the gamma-ray converter, the beam divergence may be kept at a low value. Then, the beam could be focused by a laser-driven microlens^75,76^ to a small spot size suitable for applications such as fast ignition. To make this argument more quantitative, consider a beam-focusing geometry in the context of FI as illustrated in Fig. 8. The short-pulse drives the ion beam plasma, and the electrons are cooled by a microfoil. Suppose the beam, with an initial divergence angle θ_1_, is focused by a laser-driven electric microlens placed at a distance *l*_1_ from the laser target. The lens focuses the beam at a distance *l*_2_. Assuming no angular energy correlation, the spot size is given by^77^

$$r_{s}=l_{2}(\theta_{1}+\theta_{2})\;(\delta \beta /\beta ),$$ (1)

where δβ is the ion velocity spread. For a case where the fractional ion-beam energy spread is 0.1 (the FI requirement), *l*_1_ = 200 *μ*m, and *l*_2_ = 2 cm, if θ_1_ can be kept at 1°, then *r*_s_ = 18 *μ*m, which is acceptable for FI.

### D. Gamma-ray dose

Accurate gamma-ray spectra and dose for different Trident laser targets will be presented in a separate dedicated article. For now, we can bound and estimate the spectrum as follows: First, as described above, Ta or W filters in front of the IP detectors eliminate any spectral component below 0.1 MeV. Second, we can estimate the photon energy (assuming a monoenergetic spectrum) based on the signal registered on an IP behind thick objects of different thicknesses made of high-Z materials such as W. Unfortunately, we are working near the minima (∼2 MeV) of mass absorption coefficients for such materials, which requires a detailed spectral fitting to pin down the spectrum accurately. Moreover, care must be taken to take signal ratios of nearby locations in the detector, as the intensity profile variation can easily skew the results and give unphysical results. Still, if the gamma-ray energy distribution were very broad, it would be detected based on the signal observed through different thicknesses of the material.

The intensity profile variation brings up another practical issue for an accurate estimation of the gamma-ray dose. This emission comes out in a lobe with a width similar to its pointing deviation from the laser-propagation axis, and this pointing varies from shot to shot. Therefore, given the limited size of our detectors, only shots with a sufficiently small (∼1 m) detector-source separation reliably capture the lobe maximum to provide an accurate dose measurement. It is now understood that measurements taken farther away suffer from an artificial variability induced by this effect.

With those caveats in mind, we take for example a shot with a 0.5 mm Ta converter at 200 *μ*m separation, where the PSL ratio through 1.650 versus 0.85 mm of W yields a ratio of 1.08–1.19. This corresponds to 1–2 MeV based on the NIST x-ray attenuation tables. (We have discarded unphysically high temperatures that would also match these ratios.) In a different shot, this time with a 1 mm converter separated by 150 *μ*m, the ratio of the signal through 0.7 and 1.4 cm-thick W pieces yields  ≈2  ±  0.5 MeV photon energy. Based on modeling with the MCNP code of electron transport in thick Ta disks with model energy distribution functions, these results indicate roughly similar (but hotter) beam-plasma electron distributions compared with the VPIC prediction.

With these targets and based on our calibration presented in Sec. II B 3, we have measured so far gamma-ray yield intensities *dY*_g_/*d*Ω = (1.6–1.7) × 10^13^ photons/sr in representative shots, such as (1) shot 26233, with a Ti nanofoil laser target, 68 J laser energy, a 1 mm Ta converter separated by 400 *μ*m, detector located outside the chamber at 1.45 m from the source, detecting through 0.5 cm of W; and (2) shot 26246, with a diamond/Al target, 74 J laser energy, a 1 mm Ta converter separated 130 *μ*m, detector located inside the chamber 0.91 m from the target, detecting through 0.5 cm of W. This is not the highest performance achieved on Trident, and analysis is ongoing.

It is customary to express the yield as dose that would be absorbed by a thin slab of dry air at a distance of 1 m, based on the mass-density attenuation coefficient for the appropriate energy as published by NIST. Using this convention, the dose D is given by

$$D\left[J/\text{kg}\right]\approx (\mu_{\text{air}}/\rho_{\text{air}})\;\left[m^{2}/\text{kg}\right]E_{g}\left[J\right]\;(dY_{g}/d\Omega )\left(1/d^{2}\right)\;\left[1/m^{2}\right],$$ (2)

where *μ*_air_ is the mass-density attenuation coefficient of air and *d* is the separation between the source and the detector, set to *d* = 1 m by convention. Using *E*_g_ = 2 MeV and (*μ*_air_/ρ_air_) = 4.43 × 10^−3^ m^2^/kg, the yield intensity above translates (to one significant figure) into D = 20 mSv = 2 Rads at 1 m at 2 MeV.

The figure of merit (FOM) of Rads/J in the driver is often used to estimate requirements and to compare device performance, with the implicit notion that cost scales with driver energy. Our Trident results give a FOM of 2 R/70 J = 0.03 at 2 × 10^20^ W/cm^2^ for >1 MeV photons, which represents significant progress since the reported FOM of 0.004 Rads/J for both the NOVA PW at 2 × 10^19^ W/cm^2^ (0.24 Rads/56 J) for 0.8–8 MeV photons^78^ and the Vulcan PW at 4 × 10^20^ W/cm^2^ for >0.3 MeV photons.^79^ In comparison, the rated gamma-ray performance limit of the DARHT Axis 1 relative to the electron-beam energy is 500 R/2.4 kJ = 0.2, at the price of lower resolution. The best DARHT resolution is achieved at a FOM comparable to Trident. These results are summarized in Table IV.

**TABLE IV.** Gamma-ray dose from various devices, independent of the source size.

Device	Laser intensity on target (W/cm^2^)	Dose at 1m (Rad)	Driver energy on target (J)	Rad/J
Nova PW laser	>2 × 10^19^	0.24	56	0.004
Vulcan PW laser	4 × 10^20^	0.9	230	0.004
Trident laser	2 × 10^20^	2	70	0.03
DARHT axis 1 LINAC	N.A.	500	2400	0.2

## VI. NEUTRON-GENERATION AND APPLICATIONS

Significant progress has been made in pulsed neutron-beam generation by using laser-driven deuterium beams made in the RIT regime.^26^ The directed neutron beam is generated by directing the deuterium beam into a disk of a suitable material (Be in our case). The neutrons are generated primarily by deuterium breakup/stripping nuclear reactions. The directed neutron yield exceeds by ∼4× the isotropic yield that comes from a different nuclear process.^80^ Based on the higher efficiency and average ion energy of BOA deuterium beams, multi-MeV neutron-beam yields observed at the Trident laser (*Y*_n_ ∼10^10^ neutrons in ∼1 sr and ∼1 ns) greatly exceed other laser-based attempts.^28–34^ To place our results in perspective, a summary of published results, adapted from Ref. 81, is presented in Table V, where the results at Trident are listed in the bold-faced type. Since that publication, the differential yield at Trident has increased somewhat (to 1.25 × 10^10^ n/sr), and reproducibility has greatly improved via more consistent target fabrication.

**TABLE V.** Reported neutron generation by short pulse lasers. Adapted with permission from Alejo *et al.*, Nuovo Cimento C **38**, 188 (2016).^81^

Reference	Laser energy (J)	Laser intensity (W/cm^2^)	On-axis neutron fluence (n/sr)	Fluence/laser energy (n/sr/J)
32	1	2 × 10^21^	3.0 × 10^6^	2.7 × 10^6^
29	6	2 × 10^19^	1.2 × 10^4^	2.0 × 10^3^
33	6	2 × 10^19^	4.0 × 10^5^	6.7 × 10^4^
28	69	2 × 10^19^	2.0 × 10^8^	2.9 × 10^6^
26	**80**	**8 × 10^**20**^**	**1.0 × 10^**10**^**	**1.3 × 10^**8**^**
30	127	7 × 10^20^	3.5 × 10^8^	2.8 × 10^6^
34	150	2 × 10^20^	1.0 × 10^9^	6.7 × 10^6^
31	360	2 × 10^19^	7.5 × 10^8^	2.1 × 10^6^

We present below some of what we have learned regarding spectral control of these neutron beams, as well as their suitability for neutron imaging. We also mention briefly ongoing work on extending their utility to other applications.

### A. Neutron energy spectrum

**FIG. 9.**
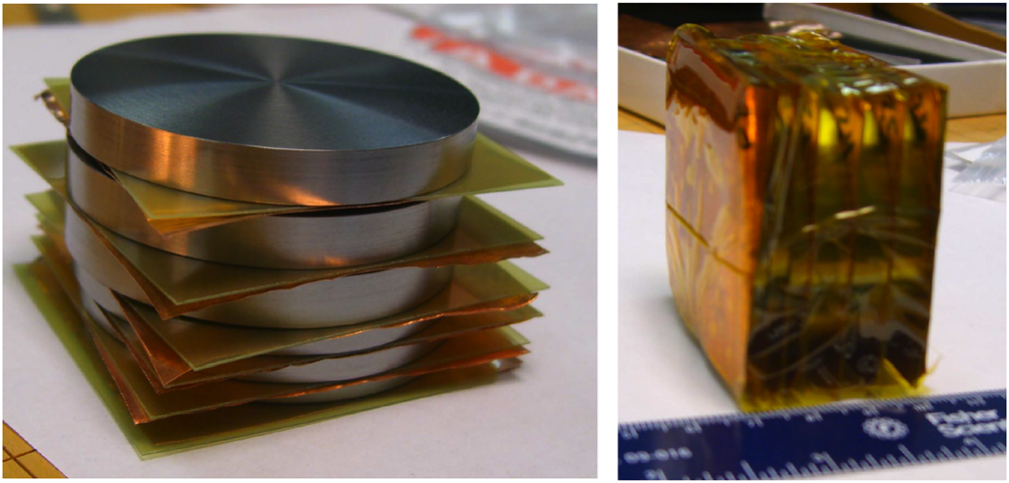
Flexible neutron converter made out of Be disks 4 cm in diameter and 0.9 cm long. The photo shows a setup where a radiochromic film is placed in between layers to study deuteron transport within the converter.

**FIG. 10.**
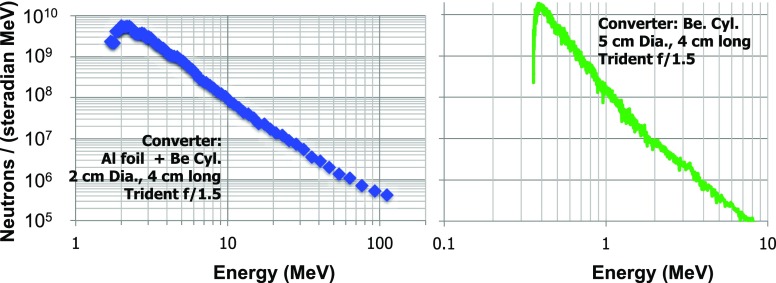
Neutron spectra for two different cylindrical Be neutron converters.

For given deuterium-beam parameters, the neutron yield and spectrum depend on the Be-converter design. This dependence has been tested experimentally with Be cylinders of different dimensions and more recently with modular converter designs composed of individual stacked disks. Figure 9 shows a photo of one such modular converter. The power of shaping the converter for neutron spectral control is shown in Fig. 10, where measured neutron-energy spectra resulting from two different converter lengths are shown. The measurements are derived from the nTOF diagnostic featuring the improved PMT mentioned in Sec. II B 2. The spectrum on the left is from the f/1.5 experimental series presented in Ref. 26, whereas the spectrum on the right is from a latter experimental series.

### B. Neutron point-projection imaging

The first laser-driven neutron image of a structured object with a short-pulse laser-driven neutron source was done using the point-projection technique at Trident and first reported in Ref. 26. The neutron-source size (which limits the resolution of the image) was measured to be ∼1 mm.^82^ For this measurement, the diagnostic was time-gated to accept neutrons in the energy range of 2.5–35 MeV. This study shows that the source size is dominated by the beam divergence angle as it propagates from the laser target to the Be converter placed 0.4 cm behind. Such a large a standoff was chosen to avoid any possibility of dispersing Be in the chamber. In a dedicated system and chamber for imaging, this standoff requirement can be relaxed.

Forward-modeling with the MCNP code was used to infer the deuterium-beam divergence from the neutron-spot size. This divergence half-angle was found to be ≈10°–15° for f/3 (compared to 9.5° for the vacuum f/number laser cone) and >35° for f/1.5 (compared to 18.5° for the laser f/number cone). A different study based on delayed nuclear reactions induced in the converter which relates the deuterium beam divergence to the observed reaction yield came to a similar conclusion for f/1.5. Such a large divergence angle may be evidence of some level of self-focusing at the higher intensity with f/1.5. Determining whether this is true and how to control it remains an interesting question for further work. Moreover, migrating the deuterium-beam generation from the conventional BOA beam to the enhanced mode described in Sec. III should greatly decrease the divergence angle and thus the neutron-source size, even with a finite laser target-converter standoff, as demonstrated with the gamma-ray source discussed in Sec. V.

**FIG. 11.**
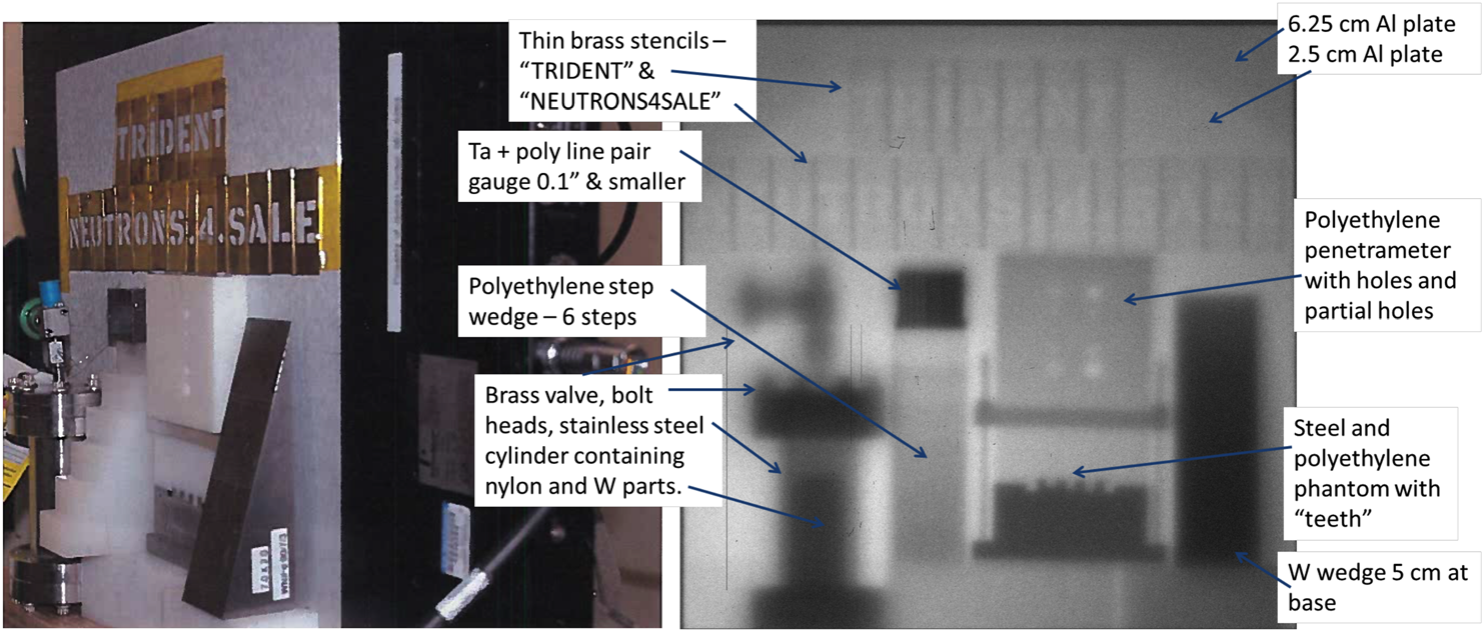
Radiography of thick objects with the laser-driven neutron-source at Trident.

In order to highlight their practical utility for imaging, a series of thick objects were imaged using our laser-driven neutron source in a similar setup as before. The Trident short-pulse laser-beam focused to f/1.5 was used to illuminate a ∼600 nm deuterated polystyrene (CD). Point projection of the thick objects on the modified 40-cm amorphous-Si computer-controlled imaging screen was done by placing the objects in the front of the screen, out of the chamber at 1.45 m from the laser target at TCC. The left side of Figure 11 shows a photo of the arrangement, while the right side of Fig. 11 shows the neutron radiograph, annotated to identify the objects. The tungsten wedge shown here is the same one shown in Fig. 6.

### C. Other applications

#### 1. Neutron-based active interrogation

Detecting the presence and transport of shielded special nuclear material fissile (SNM), especially shielded highly enriched uranium (HEU), remains a vital and difficult problem in nuclear detection^83^ and a stated US Domestic Nuclear Detection Office (DNDO) grand challenge.^84,85^ (SNM is defined by Title I of the US Atomic Energy Act of 1954 as plutonium, uranium-233, or uranium enriched in the isotope uranium-233 or uranium-235.) HEU has weak spontaneous emissions and is therefore best detected with active interrogation. Neutrons are particularly useful for characterizing SNM in nuclear safeguards and homeland security because of their ability to penetrate and to stimulate radiation emission.^86^ Current active neutron methods to measure uranium use radioisotope sources, D-T neutron generators, and, in extreme cases, linear accelerators. These have significant drawbacks. Isotropic sources are inefficient, as most neutrons do not irradiate the object being interrogated, and present significant personnel-safety challenges. Systems with fixed neutron energies cannot be optimized for variable or thick levels of shielding. High flux systems based on the conventional accelerator, reactor, or pulsed power technologies are not portable. Therefore, we are motivated to meet this global security challenge by developing a laser-based system that promises to be effective, fast, portable, affordable, and operationally safe.

In a project recently concluded at LANL, the Trident neutron beam has been used to interrogate samples of SNM and depleted U^87–89^ and was successful in all cases. Detection with delayed neutrons was demonstrated with samples of depleted U with masses of up to 4.5 kg and 990 g of 65% enriched U. Detection with prompt neutrons was demonstrated with samples of enriched U and 150 g of ^239^Pu. These results will be discussed in detail in an upcoming article. Future research plans center on enabling a practical moveable interrogation system by increasing the neutron yield efficiency and the neutron-detection efficiency.

#### 2. Bulk thermometry of dynamic materials

Another important application of laser-driven neutron sources is bulk thermometry of dynamic materials based on neutron-resonance scattering (NRS).^90^ Temperature is a fundamental thermodynamic quantity that must be measured independently for testing theories and validating equations of state of materials subjected to extreme dynamic conditions. In many cases such as in shock physics, where the timescales are too fast for embedded probes, there is no existing method to measure bulk (volumetric) temperature. One has to rely on surface pyrometry, which requires modeling for proper interpretation. Because of their penetrating power, neutrons are ideal to probe the interior of even thick, dense dynamic samples.

The NRS technique is based on detecting a notch in the spectrum of a (non-monoenergetic) neutron distribution after it traverses the object being probed. The notch results from the resonant nuclear scattering of the neutrons. NRS is routinely used in the assay of materials, but in the pioneering work in Ref. 90, the Doppler-widened resonance of a dopant in an explosively driven shocked material was interpreted as a temperature. There are known subtleties in the correct interpretation of these data (see, e.g., Ref. 91). The daunting challenge for the future is to miniaturize the setup so that it may be adopted more broadly in laboratory-scale dynamic material experiments. In such experiments, the measurement must be done in a time of <100 ns with enough neutrons to measure the widened resonance shape accurately. The laser-driven neutron source discussed above holds great promise in meeting that challenge.^92^

Recently, LANL and Darmstadt scientists demonstrated moderation of the neutron beam at Trident using a ∼7 cm block of high-density polyethylene plastic and utilized the moderated neutrons to detect the main Indium resonance at 1.4 eV.^87,93^ This important milestone will be reported in an upcoming article.

### D. For further work

Most of these applications either require or would greatly benefit from a larger neutron yield than has been demonstrated so far on Trident. Although the yield can be increased at the expense of higher laser energy, cost and system-size considerations strongly motivate maximizing efficiency to the extent possible. There remains much room for improvement in this concept, still in its infancy.

**FIG. 12.**
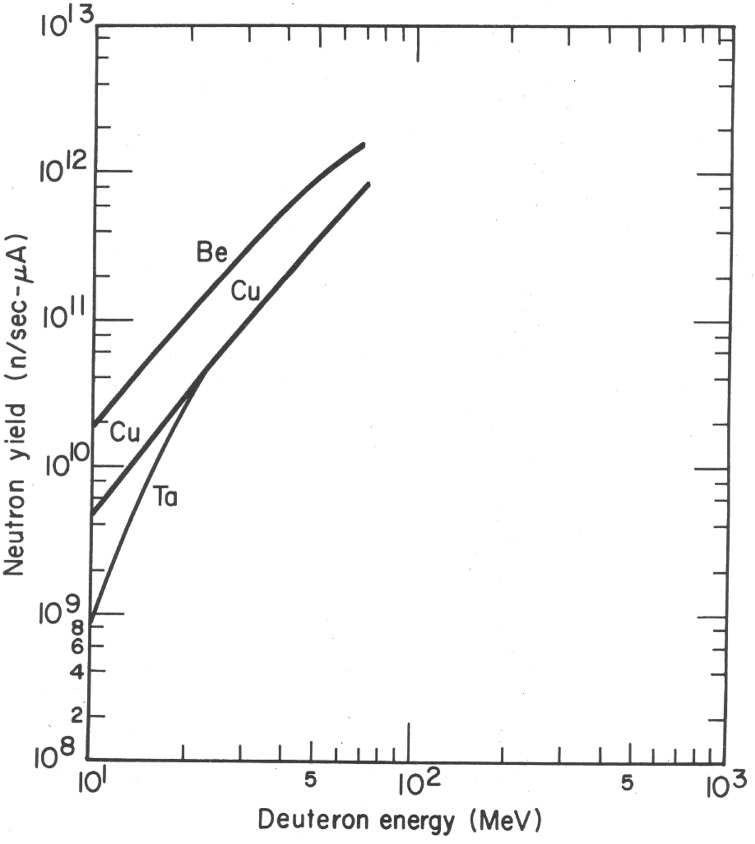
Neutron production by deuterium breakup.

An obvious direction to pursue is to increase the average energy of the laser-produced deuterium beam, ⟨*E*_D_⟩. The benefit of doing so is illustrated in Fig. 12, where the neutron-yield is plotted versus *E*_D_.^94^ The empirical dependence is fairly steep, *Y*_n_ ∼ *E*_D_^3/2^. In the RIT regime, ⟨*E*_D_⟩ ∼ *I*_L_^1/2^ at constant laser pulse length *τ*_L_ and *E*_L_.^22,48^ Therefore, one expects *Y*_n_ ∼ *I*_L_^3/4^. This is consistent with the yield increase observed on Trident on going from f/3 to f/1.5 beam focusing, which increases *I*_L_ fourfold. Therefore, it is advantageous to run with as low an f/number as possible within practical constraints.

Even at constant ⟨*E*_D_⟩, varying the deuteron energy distribution can result in significant yield variations. In a strongly skewed distribution function such as a Maxwellian, *Y*_n_ scales better with the median deuteron energy *E*_D,m_ than with ⟨*E*_D_⟩. In a Maxwellian distribution, *E*_D,m_/⟨*E*_D_⟩ = ln(2). In other words, the many more numerous slower deuterons are much less effective than the fewer faster ones in generating neutrons and completely ineffective below the 2.2 MeV reaction threshold. This is the situation with conventional BOA and TNSA deuterium beams. If one were able to extend the enhanced regime discussed in Sec. III to deuterium to obtain a quasi-monoenergetic ion beam as demonstrated for C (the same charge-to-mass ratio as deuterium), one would expect a *Y*_n_ that is higher by a factor of ≈1.7, assuming the same ⟨*E*_D_⟩. This is an approach we hope to test by using multi-layered targets incorporating deuterated plastic.

## VII. LASER AND TARGET OPTIMIZATION

The question of how to maximize *E*_i_ faced in Sec. VI D above is quite common. More generally, it is important to know how to determine the best investment of a given budget to optimize laser-driven ion-beam generation, or alternatively how to choose optimal parameters to minimize laser cost. The detailed quantitative answer for laser-driven ions in the RIT regime is understandably dependent on the ion species, whether the laser pulse is shaped in space or time, and other particulars, but the thought process is general.

**FIG. 13.**
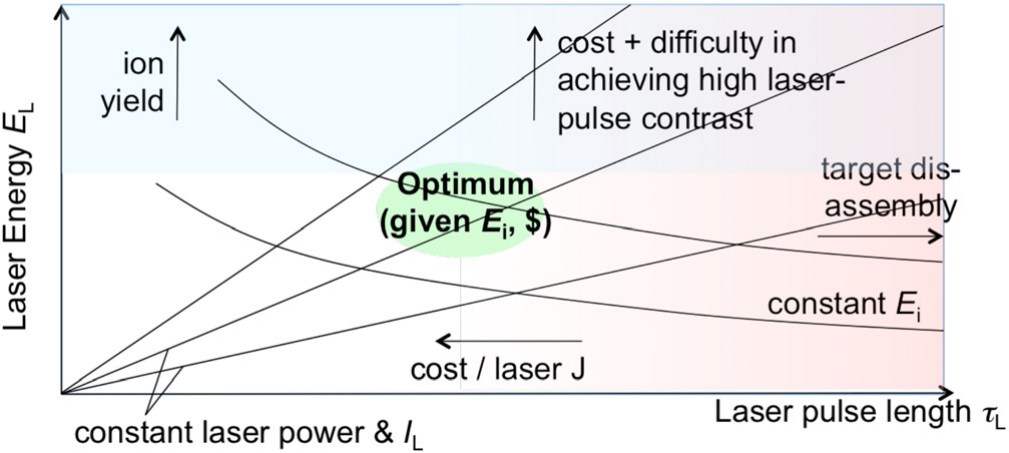
Optimization of laser parameters for laser-driven ion acceleration.

The tradeoffs involved in optimizing laser and target parameters are illustrated graphically in Fig. 13. In this figure, contours of constant laser power are straight lines converging at zero. Since the minimum laser spot size is ∼λ_L_, then maximum *I*_L_ ∼ power, and these lines are also contours of maximum laser intensity. Similarly, although the scaling with pulse length has not been investigated in detail, in general, one expects *E*_i_ ∼ *I*_L_^n^ *τ*_L_ ∼ *E*_L_^n^ τ_L_^1-n^. In the RIT regime, n ∼1/2 so that *E*_i_ ∼ *E*_L_^1/2^τ_L_^1/2^. This results in the downward-sloping profiles of constant *E*_i_ in Fig. 13. It is important to remember that one has the flexibility to operate at the same laser energy and power but at lower intensity (and lower *E*_i_) by changing the f/number of the focusing optic. It is assumed that the geometry and application will force a practical geometrical limit on how low an f/number can be used and therefore how high an intensity is achievable.

In the optimization, assuming a minimum f/number is established, it is important to remember that both higher laser energy and higher intensity (for a given energy) cost more. A higher laser energy gives higher ion yield, which pushes the optimum up. However, higher energy means higher cost and greater technological difficulty in delivering a high-contrast laser pulse, which pushes the optimum down. When considering the laser-pulse length, the severity of the laser target disassembly increases with longer pulses, which pushes one towards shorter pulses. However, cost also increases with shorter pulses, which motivates longer pulses. Taking all into account, an optimum emerges. With all these considerations, the decision can be made as follows:

• Based on the mission need, one picks the minimum *E*_i_, thus defining a contour in Fig. 13.
• If the pulse length is fixed, then the point at the *E*_i_ contour with the given pulse length defines the minimum laser energy, power, and intensity, as well as the maximum ion yield (if the laser-ion conversion efficiency is known).
• In the more interesting case of a new laser, where there may be design freedom to trade laser energy versus pulse length, one would move along the contour towards higher energy (higher ion yield) and shorter pulse and buy as much laser energy along that contour line as the budget (and the laser technology) allows. Alternatively, one also decides on the ion yield based on the mission need to set the laser energy and pulse length and thus the budget.
• The laser cost model is likely to produce a situation where for the same budget, one can end up at a higher *E*_i_ contour than the minimum specification, by choosing higher laser energy at longer pulse than if one stays on the chosen contour. In that case, one has to decide on the basis of other missions whether the extra intensity, or the extra energy, or the lower budget is more useful. If one opts for higher energy, *I*_L_ and *E*_i_ can be adjusted down by relaxing the f/number of the focusing optic and get the benefit of more robust operation.

For several specific cases we have examined, the optimum lies typically within laser pulses in the τ_L_ ≈ 0.1–1 ps range.

## VIII. SUMMARY

A large body of recent work on laser-driven ion beams and applications has been presented, carried out by interdisciplinary and multi-institutional research teams. The experimental work has been based at the LANL Trident Laser Facility. The relativistic laser-plasma simulations have been done at the LANL supercomputers using the VPIC code. The radiation-hydrodynamics simulations have been done with the Hydra code at Sandia and the HELIOS code at LANL.

With a view towards enabling applications supporting LANL science and programmatic missions, relativistic laser-plasma research at LANL has concentrated on the development of laser-driven ion beams to leverage their advantages in power density and other metrics relative to conventional beams. In collaboration with colleagues from other institutions, work at LANL has concentrated on the RIT regime, where based on modeling predictions, we have realized significant advances in performance, previously documented. In this paper (Sec. III), we discuss an extension of ion acceleration in the RIT regime where we benefit from plasma self-organization to realize a beam with a narrowed energy spread while maintaining high ion energy and conversion efficiency. This self-organization process involves self-generation of ultra-high magnetic fields and electron trapping at the rear-side of the plasma, a highly structured ion-beam distribution in phase space, and a phase-space rotation of the beam after the laser beam has passed. As before, given our minimum pulse duration and the laser-pulse energy, it is necessary to adjust the laser-target thickness to achieve relativistic transparency near the peak of the laser pulse. However, this is not enough. Accessing this regime has required operationally maintaining peak performance in laser-pulse contrast and, with some target materials, decreasing the laser-pulse energy to decrease the absolute pre-pulse level. The relative level of laser pre-pulse, which cannot be minimized further with our existing pulse-cleaning setup at Trident, affects different target materials in different ways—some are preheated more and disassemble faster than others. The self-organization dynamics do not happen if the plasma density at the onset of RIT has decreased below a certain level. Therefore, given our fixed pulse length and finite (and presently irreducible) laser-pulse contrast, the laser-pulse energy also has to be adjusted to stay within the maximum permissible for the target material. As long as these caveats are observed, the plasma self-organization process responsible for the improved performance is robust and has been demonstrated with several target materials and designs. Clever multilayer target designs are expected to allow an expansion of the performance envelope achieved to date.

These improved ion beams have been used for isochoric heating (Sec. IV), demonstrating one of our envisioned applications. This work showcases the ability we have gained to deposit volumetrically a large power density uniformly in materials at solid-density and above. In turn, this capability has enabled a new class of mix experiments that will be reported in a separate publication in the near future.

Partly motivated by gaining a better understanding of these plasmas, and partly motivated by programmatic capability gaps, we considered the utility of the relativistic electrons co-moving with the ions generated in this enhanced RIT mode. Specifically, the plasma was directed at a ∼mm disk of Ta, and the resulting emission of Bremsstrahlung gamma photons was characterized (see Sec. V). The emission comes from a small point-like source and is highly directional. This observation indicates that these electrons have a small pitch angle, consistent with the presence of a central channel in the magnetic-field structure seen in our simulations. This finding bodes well for the ability to focus these ion beams for other applications such as fast ignition. The small electron pitch angle and the small width of the plasma jet in VPIC simulation motivated us to examine the utility of this plasma as a nearly point source of gamma photons to enable higher resolution point projection radiography of thick objects. Radiography with this source was demonstrated, and a relatively high dose has been observed.

An important contribution of laser-driven ion beams in the RIT regime has been a significant performance increase in directed neutron sources (Sec. VI), which have made their practical application tantalizingly close in areas of programmatic interest. Here, we have described further progress in using modular neutron converters to modify the neutron energy distribution. We have also presented further work on point-projection imaging with these neutrons and discussed the present limits on resolution, set by the source size, and the prospects for improvement. Work on other applications being pursued has been described briefly. Our present understanding of the determinants of neutron-yield efficiency has been presented, and our strategies for improving it have been discussed.

Finally, we have discussed (Sec. VII) how ion-beam performance may be improved to benefit most of the applications discussed. Based on parameter scalings as understood presently, we have presented a methodology that may be used to properly size a laser system based on the ion-beam performance requirements.
